# The New Sydenham Society

**Published:** 1859-12

**Authors:** 


*The New Sydenham Society.*—In November, 1857, the Sydenham Society, which was organized for the purpose of giving to its members works out of print, and not likely to be reprinted, as those of Harvey, Hippocrates, or Sydenham, and some foreign works untranslated, was suspended on account of financial difficulties. The objects of this Society were admirable; and it was said at the time it was discontinued, that its lack of success was due to mismanagement. At any rate, it published about forty sterling works, some of which were of present interest, and others, the works of those whose names will always live in the profession, and who are even known to the world at large. A short time after the stoppage of the original society, however, another was organized under the name of the "New Sydenham Society," with objects like those of the old Society, with this difference, namely: to publish works of practical interest only. There is no doubt but all should know of the teachings of such men as Sydenham and Harvey, and read their books; but this is not the only question when we come to the practical matter of the organization of a publishing association. The question then arises if the profession are willing to do their duty in that regard, and will give the association sufficient support to warrant the publication of these old works. We hardly think that they will, and are disposed to regard the organization of the new association as more judicious than that of the old. The profession is poor, and members of it think they cannot buy works not of a practical character.

Not to dwell longer upon this part of the question, we can say that the old Sydenham Society had our sympathy, as we think it had that of all members of the profession taking any interest in its advancement; the regret at its suspension was as general as it was profound. We trust that the new Society will receive the cordial support of the profession, and that those whose position enables them to assist it will not neglect the opportunity. The local Secretary is Dr. C. F. Heywood, No. 60 West 20th street, New York, who is authorized to receive subscriptions and distribute the books. The subscription is one guinea, or $5 25, a year, and entitles the subscriber to the books published by the Society for that year. Through the politeness of Dr. Heywood, the first three volumes, "Diday on Infantile Syphilis," "Memoirs on Diphtheria, by Bretonneau, Trousseau," &c., and "Gooch on Diseases of Women and Children, by Ferguson," have been placed in our hands. They are handsomely printed on fair paper, and strongly bound in muslin, making three uniform, elegant volumes. It is not our purpose to go into a review of these works, the authors of which have made their names so well known in this, as well as in their own country. We copy, for the benefit of our readers, the announcement of the council, for the year 1859:—

The present volume—*Diday on Infantile Syphilis*—is the first published by the New Sydenham Society.

The following works are in the hands of the printer :—

Vol. II.—*Gooch on some of the more important Diseases of Women and Children*, and other papers. With Prefatory Essay by Dr. *Robert Ferguson*. Wood-cuts. To be ready in March.

Vol. III.—*Selected Memoirs on Diphtheria* (*Bretonneau*, *Trousseau*, *Guersent*, *Buchut*, *Daviot*, and others). With a Bibliographical Appendix. Nearly ready.

Vol. IV.—*Schrœder van der Kolk, on the Anatomy and Physiology of the Spinal Cord.* With plates.

*Schrœder van der Kolk, on the Medulla Oblongata, and on the Proximate Cause and Rational Treatment of Epilepsy.* With plates. These two volumes will be bound in one.

Vol. V.—*Clinical Memoirs on Abdominal Tumors and Intumescence.* By Dr. *Bright.* Collected and reprinted from the Guy's Hospital Reports. Edited by Dr. *Barlow.* With plates and wood-cuts.

Vol. VI.—*A Volume of Translated Modern Essays* (chiefly German), on different medical subjects. Wood-cuts.

The Council begs to announce to the members, that the first twelve months having been taken up in the organization of the Society, and the preparation of the First Year's Series of Books, it has determined that 1858–9 shall count as one year. The series of books now commenced, will, therefore, be issued for 1859, and no second subscriptions will be due until January, 1860.

A small number of extra copies of the works will be printed for the supply of those who may join the Society during the current year.

